# Sociodemographic factors and physical activity domains associated with sedentary behavior in older adults: evidence from a population-based study

**DOI:** 10.1590/0102-311XEN107925

**Published:** 2026-01-09

**Authors:** Bruno Holanda Ferreira, Tatiane Kosimenko Ferrari Figueiredo, Camila Nascimento Monteiro, Edigê Felipe de Sousa Santos, Chester Luiz Galvão Cesar, Moisés Goldbaum, Olinda do Carmo Luiz

**Affiliations:** 1 Faculdade de Medicina, Universidade de São Paulo, São Paulo, Brasil.; 2 Facultad de Ciencias de la Salud, Universidad Autónoma de Chile, Providencia, Chile.; 3 Faculdade de Saúde Pública, Universidade de São Paulo, São Paulo, Brasil.; 4 Hospital Sírio-Libanês, São Paulo, Brasil.

**Keywords:** Exercise, Health Risk Behaviors, Health Surveys, Aging, Exercício Físico, Comportamento de Risco à Saúde, Inquéritos Epidemiológicos, Envelhecimento, Ejercicio Físico, Conductas de Riesgo para la Salud, Encuestas Epidemiológicas, Envejecimento

## Abstract

Sedentary behavior is a growing public health concern due to its association with chronic diseases and functional decline, especially among older adults. This study aimed to assess the prevalence of sedentary behavior among older adults living in São Paulo, Brazil, and to analyze its associations with sociodemographic characteristics and physical activity domains. Data were collected from the *Health Survey in São Paulo City* (ISA-Capital), with 1,010 individuals aged ≥ 60 years. Sedentary behavior was assessed using the *International Physical Activity Questionnaire*, considering total sitting time (both leisure and non-leisure) and categorized as ≤ 4h/day or > 4h/day. Sociodemographic variables and physical activity domains were examined using Poisson regression models. Overall, 43.7% of participants reported sedentary behavior of > 4h/day. Multivariate analyses revealed a higher likelihood of sedentary behavior among individuals aged 80 years or older, with an increase ranging from 41% to 58% compared to those aged 60-69 years. Men were 23% more likely to report sedentary behavior than women. Participants with four or more years of education had a 29% to 64% higher likelihood compared to those with up to three years. Individuals who self-identified as Indigenous or others had a 38% to 41% greater likelihood compared to white participants. Engaging in less than 150 min/week of commuting, domestic, and total physical activity was associated with a 41% to 67% increase in sedentary behavior. These results highlight sociodemographic disparities and the influence of specific physical activity domains on sedentary behavior among older adults, reinforcing the need for targeted public health interventions.

## Abstract (pt)

O comportamento sedentário é uma preocupação crescente de saúde pública devido à sua associação com doenças crônicas e o declínio funcional, especialmente em idosos. Este estudo teve como objetivo avaliar a prevalência de comportamento sedentário entre idosos residentes em São Paulo, Brasil, bem como analisar sua associação com características sociodemográficas e domínios de atividade física. Os dados foram coletados da *Inquérito de Saúde do Município de São Paulo* (ISA-Capital), com 1.010 indivíduos com idade ≥ 60 anos. O comportamento sedentário foi avaliado usando o *Questionário Internacional de Atividade Física*, considerando o tempo total sentado (lazer e não lazer) e categorizado como ≤ 4 ou > 4 horas/dia. As variáveis sociodemográficas e os domínios de atividade física foram examinados usando modelos de regressão de Poisson. No geral, 43,7% relataram comportamento sedentário de > 4 horas/dia. As análises multivariadas revelaram uma maior probabilidade de comportamento sedentário entre indivíduos com 80 anos ou mais, com um aumento variando de 41% a 58% quando comparados com aqueles com idade entre 60-69 anos. Os homens tiveram 23% mais chances a relatar comportamento sedentário do que as mulheres. Os participantes com quatro ou mais anos de escolaridade apresentaram uma probabilidade 29% a 64% maior quando comparados com aqueles com até três anos. Os participantes que se autoidentificaram como indígenas ou outros apresentaram uma probabilidade 38% a 41% maior quando comparados com os participantes brancos. Praticar menos de 150 minutos/semana de deslocamentos, atividades domésticas e atividades físicas totais foi associado a um aumento no comportamento sedentário, variando de 41% a 67%. Esses resultados destacam disparidades sociodemográficas e a influência de domínios específicos de atividade física no comportamento sedentário entre os idosos, reforçando a necessidade de intervenções direcionadas em saúde pública.

## Abstract (es)

El sedentarismo es un problema de salud pública cada vez más preocupante debido a su relación con enfermedades crónicas y deterioro funcional, especialmente en personas mayores. El objetivo de este estudio fue evaluar la prevalencia del sedentarismo entre las personas mayores que viven en São Paulo (Brasil) y analizar su relación con las características sociodemográficas y los espacios de actividad física.Los datos se recopilaron a partir de la *Encuesta de Salud en el Municipio de São Paulo* (ISA-Capital), con 1.010 personas de ≥ 60 años. El comportamiento sedentario se evaluó mediante el *Cuestionario Internacional de Actividad Física*, teniendo en cuenta el tiempo total que se pasa sentado (porocioynoporocio) y se clasificó en ≤ 4 o > 4 horas/día. Las variables sociodemográficas y los espacios de actividad física se examinaron utilizando modelos de regresión de Poisson. El 43,7% declaró tener un comportamiento sedentario de más de 4 horas al día. Los análisis multivariantes revelaron una mayor probabilidad de comportamiento sedentario entre las personas de 80 años o más, con un aumento de entre el 41% y el 58% en comparación con las personas de entre 60 y 69 años. Los hombres tenían un 23% más de probabilidades de declarar un comportamiento sedentario que las mujeres; aquellos con cuatro o más años de escolaridad presentaban una probabilidad de sedentarismo entre un 29% y un 64% mayor en comparación con aquellos con hasta tres años. Los individuos que se auto identificaron como indígenas u otros tenían entre un 38% y un 41% más de probabilidades de tener una conducta sedentaria en comparación con los participantes blancos. Realizar menos de 150 minutos semanales de actividad física (en desplazamientos, actividades domésticas y en general) se asoció con un aumento del sedentarismo, que osciló entre el 41% y el 67%. Estos resultados resaltan las disparidades sociodemográficas y la influencia de espacios específicos de actividad física en el sedentarismo entre los adultos mayores, lo quere fuerza la necesidad de intervenciones específicas de salud pública.

## Introduction

The global older adult population has been rapidly increasing due to advancements in the treatment and management of various diseases. It is estimated that, by 2050, approximately 22% of the world’s population will be aged 60 years or older ^1^, whereas in Brazil, this proportion is expected to reach 14% by 2035 ^2^. In addition to this significant demographic shift, a substantial number of older adults live with chronic noncommunicable diseases such as cardiovascular diseases, diabetes, cancer, and dementia, as well as physical disabilities, frailty, and psychological disorders ^2^, representing a growing challenge for healthcare and social assistance systems ^3^.

Another factor influenced by technological, social, and environmental transformations in recent years is the change in lifestyle patterns, particularly the adoption of unhealthy behaviors. Increasingly, people spend a considerable amount of their waking time sitting or reclining. These waking activities, performed with an energy expenditure ≤ 1.5 metabolic equivalents, are defined as sedentary behavior ^4^ and differ from physical inactivity, which refers to a lack of moderate or vigorous physical activity. In developed countries, approximately 60% of older adults report spending four or more hours per day sitting ^5^, whereas in Brazil, this estimate is 31.2% ^6^. The threshold of four hours per day as an indicator of high sedentary behavior has been established in previous observational studies ^5^ ^,^ ^7^. In light of this behavior, research in recent years has revealed that sedentary behavior has become a global public health issue. Although there is no universally established threshold for sedentary behavior, evidence suggests that sitting for four or more hours per day is associated with an increased risk of all-cause mortality ^8^. Sitting ≥ 4h/day has been linked to higher mortality risk in older adults, metabolic syndrome, increased waist circumference, overweight and obesity ^9^, functional disability ^10^, and multimorbidity ^6^.

The literature has indicated that, in addition to poor health and health inequalities ^11^, socioeconomic position is a key determinant of sedentary behavior in both high-income ^12^ ^,^ ^13^ and low- and middle-income countries ^14^, including Brazil ^15^. In developed countries, socioeconomic disadvantage among older adults has been associated with greater exposure to sedentary behavior ^16^. Given these findings, studies have proposed strategies to reduce sedentary behavior, including engaging in physical activity ^7^ ^,^ ^17^.

Many studies have explored the relationships between leisure-time physical activity, overall physical activity, and sedentary behavior, showing that sedentary behavior is significantly and positively associated with insufficient physical activity and prolonged television viewing in both sexes. Moreover, replacing sitting time with physical activity has been associated with higher quality of life scores and greater subjective well-being ^17^ ^,^ ^18^ ^,^ ^19^. However, a gap remains concerning other physical activity domains - such as transportation, domestic, and occupational settings - and the influence of sociodemographic differences on these associations should be examined while accounting for potential confounding factors.

As suggested by the literature, identifying current levels of sedentary behavior is crucial for understanding the magnitude of the problem and for informing public policies aimed at implementing interventions that promote healthy habits, such as encouraging physical activity and reducing sedentary behavior ^20^ ^,^ ^21^ ^,^ ^22^. This study aims to estimate the prevalence of sedentary behavior among older adults living in the city of São Paulo, Brazil, and to analyze its relationship with socioeconomic characteristics and physical activity domains.

## Methods

This population-based cross-sectional study was conducted in 2015 using data from the *Health Survey in São Paulo City* (ISA-Capital). The study sample represents the urban population of São Paulo, a city located in southeastern Brazil. São Paulo has over 12 million residents, approximately 11.6% of whom are aged 60 and older ^23^. It is the largest and most economically significant city in Brazil and the fourth most populous metropolis in the world.

ISA-Capital aimed to evaluate the population’s health status according to living conditions, focusing on lifestyle indicators and chronic diseases. The sampling employed a complex and probabilistic methodology, with randomized selections of census sectors and households. Two domains were considered: geographic via regional health districts (Central-West, East, North, Southeast, and South) and demographic (male and female older adults). The sample calculation was based on 980 individuals, with 162 to 234 planned interviews per district, enabling the estimation of proportions of 0.50, a sampling error of 0.10, a 95% confidence interval (95%CI), and a design effect of 1.524.

Each randomly selected household was visited at least three times, resulting in a response rate of 0.76. Among eligible residents, the individual response rate was 0.74. After applying sampling weights, the ISA-Capital data represented approximately 1,604,869 individuals aged 60 years or older residing in permanent private households within the city of São Paulo. For this study, bedridden individuals, wheelchair users, homeless individuals, and residents of institutions were excluded. A detailed description of the 2015 ISA-Capital sampling plan is provided in another publication ^24^.

The questionnaire was divided into thematic blocks, with most questions being closed-ended with predefined response options. Informed consent was obtained from all participants after a complete explanation of the study’s purpose and procedures, and all participants signed a free and informed consent form. The study complies with *Resolution n. 466/2012* of the Brazilian National Health Council (CNS, acronym in Portuguese).

Sedentary behavior, the dependent variable, was assessed using the long version of the *International Physical Activity Questionnaire* (IPAQ), which has been validated for evaluating sedentary behavior and physical activity levels ^25^ ^,^ ^26^ and has been used in other studies with ISA-Capital data ^27^ ^,^ ^28^ ^,^ ^29^ ^,^ ^30^. Participants were asked: “How much time do you spend sitting on a weekday?” and “How much time do you spend sitting on a weekend day?”.

Average daily sitting time was calculated as a weighted mean: weekday sitting times were multiplied by five, weekend sitting times by two, and the total divided by seven. 

Sedentary behavior was categorized as follows: (a) < 4h/day and (b) ≥ 4h/day. While other studies have employed different cut-offs - such as < 3h/day, 6h/day 6 or 8h/day ^31^, particularly in younger or mixed-age populations - the < 4h/day threshold was considered more appropriate for older adults in this study. In our sample, the mean daily sitting time was 4.2h/day (median: 3.6h/day; interquartile range: 2-6h/day), with the 4h/day cut-off lying near the median, supporting its suitability for distinguishing lower and higher sedentary time. Moreover, this threshold is supported by strong epidemiological evidence and aligns with previous research in this age group, which has shown that sitting for 4h/day or more is associated with an increased risk of all-cause mortality ^7^ ^,^ ^32^.

The independent variables explored in this study included different domains of physical activity (leisure-time, domestic, commuting, and occupational) and total physical activity. Scores for each domain were calculated according to IPAQ guidelines (https://sites.google.com/view/ipaq/home), multiplying vigorous activities by two ^29^ ^,^ ^30^. Participants were classified into two categories: 0-149min/week and ≥ 150min/week of moderate-to-vigorous physical activity within a specific domain. This criterion was applied separately to each domain as an analytical strategy and, therefore, does not indicate whether participants meet overall physical activity recommendations, which are based on total activity accumulated across all domains. This approach is consistent with methods used in previous studies ^29^ ^,^ ^30^ ^,^ ^33^ ^,^ ^34^. Other variables analyzed included age range in complete years (60-69, 70-79, ≥ 80) ^29^; sex (male or female); self-reported race/ethnicity (white or yellow, black or mixed-race, Indigenous or other); marital status (with a partner or without a partner); and years of education completed (0-3, 4-7, 8-11, 12 or more). Additional variables, such as back pain (yes/no), smoking (yes/no), current alcohol use (yes/no), and body mass index (underweight, adequate weight, overweight, obesity) ^35^, were also included.

### Statistical analysis

Descriptive analysis included estimating prevalence rates and their respective 95%CI for sociodemographic and behavioral characteristics. Prevalence ratios (PR) and 95%CI were estimated using crude Poisson regression with robust variance to identify potential associations between sedentary behavior and sociodemographic characteristics or physical activity domains. Poisson regression was selected given the cross-sectional design and the high prevalence of the binary outcome (> 10%), providing more conservative prevalence estimates with confidence intervals ^36^. Participants with incomplete information were treated as missing data.

Two multiple Poisson regression models were fitted to examine the association between sedentary behavior and independent variables: (a) adjusted for sociodemographic variables (age range, education, gender, and race/ethnicity), which are widely recognized in the literature as key determinants of sedentary behavior ^31^ ^,^ ^37^; and (b) adjusted for sociodemographic variables plus back pain, smoking, current alcohol use, and body mass index (BMC). The second adjustment accounted for additional health-related and lifestyle factors that may influence sedentary behavior, providing a more comprehensive understanding of associations ^37^ ^,^ ^38^ ^,^ ^39^. Multicollinearity was assessed using the variance inflation factor (VIF) for all independent variables in each regression model; all VIF values were below 1.5, indicating no evidence of multicollinearity. Including more variables in the model does not necessarily increase the precision of estimates. A 5% significance level was adopted for all tests.

All analyses were performed using the Stata software (https://www.stata.com), which incorporates aspects of the complex sampling design − strata, clusters, and weights − via the survey module.

## Results

Table 1 presents the characteristics of the study participants (n = 1,010). Most participants were aged 60-69 years, female, with a partner, had low education levels, and self-identified as white and Yellow. Additionally, a higher prevalence of older adults reported engaging in less than 150min/week of leisure-time, occupational, commuting, and domestic physical activity. Conversely, a higher proportion achieved ≥ 150min/week of total physical activity. Most participants reported no back pain and spent, on average, four hours or less per day engaging in sedentary behavior.

**Table 1:** Distribution of sociodemographic characteristics, lifestyle habits, and sedentary behavior. *Health Survey in São Paulo City* (ISA-Capital).

Parameters and categories	%	95%CI
Age range (years)		
60-69	57.6	53.5-61.7
70-79	28.3	24.6-32.1
80 or more	14.1	11.3-16.9
Sex		
Female	59.8	57.0-62.7
Male	40.2	37.3-43.0
Marital status *		
With partner	52.5	48.4-56.6
Without partner	47.5	43.4-51.6
Education (years) **		
0-3	47.3	42.1-52.5
4-7	15.2	12.7-17.6
8-11	18.1	15.6-20.6
12 or more	19.4	14.8-24.1
Race/Ethnicity ***		
White and yellow	68.4	64.0-72.8
Black and mixed-race	27.4	23.1-31.7
Indigenous and others	4.2	2.7-5.7
Leisure-time physical activity (min/week)		
≥ 150	16.6	13.4-19.8
0-149	83.4	80.2-86.6
Occupational physical activity ^#^ (min/week)		
≥ 150	12.2	9.7-14.7
0-149	87.8	85.3-90.3
Commuting physical activity ^##^ (min/week)		
≥ 150	15.5	12.4-18.6
0-149	84.5	81.4-87.6
Domestic physical activity ^###^ (min/week)		
≥ 150	47.9	44.2-51.7
0-149	52.1	48.3-55.8
Total physical activity ^§^ (min/week)		
≥ 150	69.2	65.3-73.1
0-149	30.8	26.9-34.7
Back pain ^§§^		
No	58.2	54.1-62.3
Yes	41.8	37.7-45.9
Sedentary behavior (hours/day)		
≤ 4	56.3	52.4-60.2
> 4	43.7	39.8-47.6
Smoking ^§§§^		
No	87.9	85.5-90.4
Yes	12.1	9.6-14.5
Current alcohol use ^†^		
No	72.9	68.2-77.6
Yes	27.1	22.4-31.8
Body mass index ^††^		
Underweight	20.9	17.9-23.9
Adequate weight	45.0	41.3-48.7
Overweight	13.1	10.5-15.6
Obesity	21.1	18.2-23.9

95%CI: 95% confidence interval.

Note: missing data were treated as excluded from the analyses.

* 3 missing;

** 5 missing;

*** 7 missing;

^#^ 8 missing;

^##^ 2 missing;

^###^ 4 missing;

^§^ 15 missing;

^§§^ 2 missing;

^§§§^ 6 missing;

^†^ 6 missing;

^††^ 46 missing.

Table 2 presents the distribution and regression analysis of sedentary behavior among older adults, categorized by sociodemographic characteristics and physical activity domains. The crude regression analysis identified a significant association between sedentary behavior and sex, education, race/ethnicity, and physical activity in the commuting, domestic, and total domains (p < 0.05).

**Table 2:** Distribution and regression analysis of sedentary behavior among older adults according to sociodemographic characteristics and physical activity domains. *Health Survey in São Paulo City* (ISA-Capital).

Parameters and categories	≤ 4h/day		> 4h/day *		Crude PR (95%CI)	Adjusted PR (95%CI) **	Adjusted PR (95%CI) ***
	%	95%CI	%	95%CI			
Age range (years)							
60-69	58.7	53.4-63.8	41.3	36.2-46.6	1.00	1.00	1.00
70-79	55.2	48.2-62.0	44.9	38.1-51.9	1.09 (0.90-1.31)	1.16 (0.96-1.40)	1.15 (0.95-1.38)
80 or more	49.2	40.2-58.2	50.8	41.8-59.8	1.23 (0.99-1.53)	1.41 (1.11-1.79)	1.58 (1.23-2.03)
Sex							
Female	60.5	56.0-64.9	39.5	35.1-44.0	1.00	1.00	1.00
Male	50.1	44.0-56.3	49.9	43.7-56.0	1.26 (1.08-1.48)	1.23 (1.05-1.44)	1.23 (1.06-1.43)
Marital status							
With partner	54.8	48.7-60.7	45.2	39.3-51.3	1.00	1.00	1.00
Without partner	58.0	52.9-63.0	42.0	37.0-47.1	0.93 (0.77-1.12)	0.99 (0.81-1.20)	0.99 (0.81-1.20)
Education (years)							
0-3	62.1	56.6-67.2	37.9	32.8-43.4	1.00	1.00	1.00
4-7	52.6	42.6-62.5	47.4	37.5-57.4	1.25 (0.96-1.62)	1.26 (0.98-1.62)	1.29 (1.01-1.65)
8-11	58.7	50.9-66.1	41.3	33.9-49.1	1.09 (0.84-1.41)	1.13 (0.87-1.47)	1.17 (0.90-1.51)
12 or more	42.9	32.1-54.3	57.1	45.7-67.9	1.51 (1.17-1.94)	1.54 (1.20-1.99)	1.64 (1.25-2.14)
Race/Ethnicity							
White and yellow	53.9	48.7-59.0	46.1	41.0-51.3	1.00	1.00	1.00
Black and mixed-race	63.8	57.5-69.7	36.2	30.4-42.5	0.79 (0.63-0.98)	0.87 (0.70-1.07)	0.87 (0.70-1.07)
Indigenous and others	43.8	27.4-61.6	56.2	38.4-72.6	1.22 (0.88-1.69)	1.38 (1.01-1.88)	1.41 (1.02-1.93)
Leisure-time physical activity (min/week)							
≥ 150	57.5	47.8-66.6	42.5	33.4-52.2	1.00	1.00	1.00
0-149	56.1	51.8-60.3	43.9	39.7-48.2	1.03 (0.81-1.31)	1.10 (0.86-1.41)	1.07 (0.84-1.35)
Occupational physical activity (min/week)							
≥ 150	55.6	46.0-64.8	44.4	35.2-54.0	1.00	1.00	1.00
0-149	56.1	52.0-60.2	43.9	39.8-48.0	0.99 (0.79-1.23)	0.98 (0.78-1.23)	0.97 (0.76-1.22)
Commuting physical activity (min/week)							
≥ 150	70.6	62.6-77.5	29.4	22.5-37.4	1.00	1.00	1.00
0-149	53.7	49.5-57.8	46.3	42.2-50.5	1.57 (1.22-2.03)	1.64 (1.25-2.15)	1.56 (1.19-2.03)
Domestic physical activity (min/week)							
≥ 150	68.7	62.6-74.2	31.3	25.8-37.4	1.00	1.00	1.00
0-149	44.8	39.8-49.9	55.2	50.1-60.2	1.76 (1.43-2.18)	1.67 (1.32-2.11)	1.64 (1.31-2.05)
Total physical activity (min/week)							
≥ 150	61.9	57.0-66.5	38.1	33.5-43.0	1.00	1.00	1.00
0-149	42.4	36.1-48.9	57.6	51.1-63.9	1.51 (1.29-1.77)	1.47 (1.23-1.76)	1.41 (1.18-1.68)

95%CI: 95% confidence interval; PR: prevalence ratio.

Note: in bold, p-value ≤ 0.05 means statistical difference.

* Event of interest;

** Adjusted for age range; sex, education, and race/skin color;

*** Adjusted for age range; sex; education; race/skin color; back pain, smoking; current alcohol use, and body mass index.

In the adjusted models, compared with older adults aged 60-69 years, those aged 80 years or older had a 41% (Model A) to 58% (Model B) greater likelihood of engaging in sedentary behavior. Men were 23% (Model A and B) more likely to report sedentary behavior than women.

Compared with those with up to three years of education, individuals with four to seven years of education had a 29% greater likelihood of sedentary behavior (Model B), while those with 12 or more years of education had a 54% (Model A) to 64% (Model B) greater likelihood. Participants who self-identified as Indigenous or others were 38% (Model A) to 41% (Model B) more likely to exhibit sedentary behavior than those who self-identified as white and yellow. No significant differences were observed for marital status (p > 0.05).

In the commuting physical activity domain, older adults who engaged in less than 150min/week had a 64% (Model A) and 56% (Model B) likelihood of sedentary behavior compared with those who achieved ≥ 150min/week. Similar results were found for the domestic domain, with a 67% (Model A) to 64% (Model B) increase, and for total physical activity, with a 47% (Model A) to 41% (Model B) greater likelihood of sedentary behavior. No significant differences were detected in the leisure or occupational domains (p > 0.05).

## Discussion

A high prevalence of older adults spending four or more hours sitting per day was estimated. Factors associated with a greater likelihood of sedentary behavior included being 80 years or older, male, self-identifying as Indigenous or others, having four or more years of education, and engaging in up to 150min/week of commuting, domestic, and total physical activity compared to their peers.

Few studies address the prevalence of sedentary behavior among older adults in Brazil ^28^ and worldwide ^5^ ^,^ ^40^. In this study, four out of ten older adults living in São Paulo spent, on average, four or more hours sitting per day. This finding aligns with other national ^20^ ^,^ ^28^ and international research ^5^. In São Paulo, Rocha et al. ^28^ investigated sedentary behavior in a population aged up to 65 years and found a median sitting time of 180 minutes per day. Interestingly, the highest ever average daily sitting time, around 240 minutes per day, was observed in the younger age group (20-29 years) ^28^, indicating that sedentary behavior is common across different age groups ^20^.

This study has identified a greater prevalence of sedentary behavior among older adults aged 80 years or older, which is consistent with previous findings ^40^ ^,^ ^41^ ^,^ ^42^. This high prevalence may be partly explained by increased mobility difficulties, loss of muscle mass (sarcopenia), chronic pain, and other health conditions common in this age group, which can limit physical activity and lead to longer periods of sitting or lying down. Conversely, a review revealed that individuals aged 74 years or younger used computers more frequently ^5^, reflecting technology-related habits among younger cohorts and suggesting a potential increase in sedentary behavior in future generations of older adults.

A relationship between sex and sedentary behavior was observed. Older men were more likely to spend time sitting compared to older women, a pattern similar to that reported in other studies ^5^ ^,^ ^20^ ^,^ ^31^ ^,^ ^41^. This difference may be partly explained by sociocultural factors, such as the greater involvement of older women in domestic tasks (e.g., cooking, cleaning, laundry), which require light physical effort and consequently reduce sedentary time ^31^. In contrast, older men may engage more frequently in sedentary leisure activities such as watching television. These gendered behavioral patterns reflect traditional social roles and responsibilities and suggest that interventions aimed at reducing sedentary behavior should consider such dynamics. However, further research is needed to better understand these complex relationships.

Global evidence has shown a relationship between educational attainment and sedentary behavior among adults ^20^ ^,^ ^31^ and older adults ^40^ ^,^ ^43^. Research suggests that adults with higher education levels in more developed countries are more likely to occupy sedentary jobs, rely on motorized transportation, and engage in electronic entertainment and occupation convenience devices ^20^, thereby increasing the risk of adopting unhealthy behaviors. Notably, evidence highlights retirement as a critical transition and an opportunity for health promotion and reducing health inequalities ^16^. This stage often involves substantial changes in daily routines, which may either increase sedentary time due to reduced work-related activity or foster greater participation in leisure-time physical activity. Policies addressing health disparities in adulthood should prioritize creating supportive environments − including community programs, accessible facilities, and opportunities for social engagement − for older adults to replace sedentary behavior with meaningful and enjoyable activities ^16^.

In middle-income countries such as Brazil, the relationship between education level and sedentary behavior may follow a different pattern. Older adults with higher education may have greater access to sedentary leisure activities, such as prolonged screen use or motorized transportation, which may contribute to higher sedentary time. Conversely, those with lower education levels are more likely to engage in physically demanding domestic or informal work, potentially reducing sedentary time. These differences underscore the importance of considering socioeconomic and cultural contexts when interpreting the association between education and sedentary behavior across diverse populations.

A greater likelihood of sedentary behavior was found among individuals who self-identified as Indigenous or other racial groups. This finding aligns with a previous study conducted in Brazil, which identified higher sedentary behavior among individuals who self-identified as black, mixed-race, or Indigenous ^44^ but contrasts with results from studies conducted in the United States ^45^. Such differences may be linked to environmental and cultural barriers that hinder opportunities for behavioral changes among ethnic minorities. These findings underscore the need for health policy interventions tailored to the specific characteristics of individuals who self-identify as Indigenous or other.

This study also identified a relationship between physical activity in the commuting, domestic, and total domains and sedentary behavior. The findings for overall physical activity are consistent with previous research ^20^ ^,^ ^31^. In a study involving adults aged 35-74 years, Pitanga et al. ^31^ reported that both leisure-time and commuting physical activity were inversely associated with sedentary behavior. However, evidence on the association between physical activity in the commuting and domestic domains and sedentary behavior remains scarce, particularly among older adults.

Some limitations of this study must be acknowledged. Due to its cross-sectional design, causality and the direction of associations cannot be established. As noted in the physical activity literature ^46^ ^,^ ^47^, the identified variables should be interpreted as correlates rather than determinants. Nevertheless, the study makes a significant contribution by identifying variables that impact health and generating hypotheses for future research. Another limitation is the exclusion of institutionalized populations residing in São Paulo. Additionally, no clinical or laboratory tests were used to directly measure physical activity, sedentary behavior, or health conditions. Although devices such as accelerometers, pedometers, and inclinometers, provide more precise measurements of movement and posture, their use is often costly and time-consuming, limiting feasibility in extensive population-based studies. This study’s strength lies in its use of the long version of the IPAQ, a validated and widely used instrument ^48^ that enables the independent evaluation of various physical activity domains and facilitates comparisons across studies. However, as with any self-report instrument, the IPAQ is subject to recall bias and potential over- or underestimation of activity levels, which may affect data accuracy. These limitations are well-documented in the literature and should be considered when interpreting the findings. Finally, as the questionnaire refers to the previous week, participants are more likely to recall their physical activity and sedentary behavior.

This study estimated sedentary behavior among older adults residing in Brazil’s largest city, revealing a high prevalence of individuals spending, on average, four or more hours sitting per day. This highlights the need to reinforce monitoring efforts to reduce sedentary time. Promoting physical activity − even light activities such as casual, slow-paced walking ^49^ and campaigns such as “*Stand Up, Sit Less, Move More*” ^50^ (p. 374) − should target older adults, regardless of their health limitations.

Moreover, older adults aged 80 years or older, males, those with higher educational attainment, self-identifying as Indigenous or others, and those engaging in up to 150min/week of commuting, domestic, and total physical activity were more likely to engage in sedentary behavior. This suggests that sociodemographic and behavioral factors should be considered when planning public policies aimed at reducing sedentary behavior.

## Data Availability

The research data are available upon request to the corresponding author.
